# Ventilator associated pneumonia and transfusion, is there really an association? (the NAVTRA study)

**DOI:** 10.1186/1471-2466-6-18

**Published:** 2006-07-25

**Authors:** David Yepes, Bladimir Gil, Olga Hernandez, Rodrigo Murillo, Marco Gonzalez, Juan Pablo Velasquez

**Affiliations:** 1 Department of critical care, Clinica Universitaria Bolivariana and Clinica CES, Department of epidemiology, University CES, Medellín, Colombia; 2 Department of critical care, Clínica Las Américas, Clínica Medellín, Grupo de Investigación en Cuidado Crítico, Universidad Pontificia Bolivariana, Department of epidemiology, University CES, Medellín, Colombia; 3 Department of critical care, Clinica Medellín, Medellín, Colombia; 4 Department of critical care, Clinica Medellin, Hospital Pablo Tobon Uribe, Grupo de Investigacion en cuidado critico, Universidad Pontificia Bolivariana, Medellín, Colombia; 5 Department of critical care, Clinica Medellín, Grupo de Investigacion en cuidado critico, Universidad Pontificia Bolivariana, Medellín, Colombia; 6 Division medical and critical care medicine, Surgical Critical Care unit, University Nueva Granada, Hospital Militar, Bogota, Colombia

## Abstract

**Background:**

Anemic syndrome is a frequent problem in intensive care units. The most probable etiology is the suppression of the erythropoietin response due to the direct effects of cytokines, as well as frequent blood sampling.

Transfusions are not free of complications, therefore transfusion reactions are estimated to occur in 2% of the total packed red blood cells (pRBCs) transfused.

In the past several years, several trials had tried to compare the restrictive with the more liberal use of transfusions, and they were found to be equally effective.

Nosocomial pneumonia is the most common nosocomial infection in intensive care units; the prevalence is 47% with an attributive mortality of 33%.

There are multiple risk factors for the development of nosocomial pneumonia. Colonization of the upper airways is the most important pathophysiological factor but there are other factors implicated like, sedation techniques, inappropriate use of antibiotics and recumbent positioning.

A secondary analysis of the CRIT study describes transfusion therapy and its practices in the United States. They found that transfusion practice is an independent risk factor for the development of nosocomial pneumonia.

**Methods:**

This is a multicenter, prospective cohort study in different intensive care units in Colombia. A total of 474 patients were selected who had more than 48 hours of mechanical ventilation.

The primary objective is to try to demonstrate the hypothetical relationship between the use of transfusions and nosocomial pneumonia.

Secondly, we will try to determine which other factors are implicated in the development of pneumonia in intensive care units and describe the incidence of pneumonia and transfusion practices.

**Discussion:**

Ventilator associated pneumonia is a primary problem in the intensive care unit, multiple factors have been associated with its presence in this study we try to explore the possible association between pneumonia and transfusion, describe all other factors associated with this, and the possible association with other nosocomial infections.

## Background

Anemia is a frequent problem in intensive care units. The most probable etiology is the suppression of the erythropoietin response due to the direct effects of cytokines, as well as frequent blood sampling [1].

In the United States it is estimated that 3,500 transfusions are given daily, around 1.25 million transfusions throughout the year, and 73% of patients that stay in an intensive care unit for more than 7 days are transfused [2], with pretransfusion average hemoglobin of 8.4 g/dL

One of the most alarming risks of anemia is lost of capacity to transport oxygen. Nevertheless, animal studies have found tolerance levels of hemoglobin as low as 3 g/dL, as well as a good tolerance of hemodilution, electrocardiographic changes and ventricular function, at these levels [3,4].

Elderly patients with coronary artery disease, cerebrovascular disease and pulmonary disease are groups considered intolerant to important decreases in the levels of hemoglobin. A common use of transfusion practice in hospitalized patients is to maintain hemoglobin levels within normal limits. However, there is no evidence that confirms these findings [5].

Transfusion practice involves multiple health risk due to its inadequate use. Adverse effects are frequent and can present in up to 2% of transfusions including infrequent diseases as hepatitis C and HIV [6] as well as immune-mediated reactions, with an incidence of 1 in 1.000.000 fatal hemolytic reactions [7]. Other more commonly seen are fluid overload along with noninfectious complications such as non- hemolytic, febrile reactions as well as allergic reactions.

TRALI is a non-cardiogenic type of pulmonary edema associated with transfusions, classified as a serious complication, which has been the cause of 45 deaths within 1992 – 2002. Its pathogenesis is the entrapping of leukocytes in the pulmonary microcirculation with an increase in pulmonary permeability [8]. Immunomodulation-associated transfusion which was used in the past to improve survival in transplanted patients has not been systematically studied. An association between the recurrence of cancer, the presence of nosocomial infections and the use of transfusions has been reported [9]. There is evidence that stored leukocytes found in a unit of blood could produce cytokines that interfere with the immune function. A transfusion introduces new antigens as Histocompatibility antigens (HLA) which interact with native T-cells as well as molecules called co stimulators that can cause a suppression of immune response [10].

Microquimerism has been proposed as another mechanism of immunomodulation-associated transfusions. Although, HLA compatibility between donor and receiver can result in the persistence of leukocytes from the donor and antigen presenting cells causing a decrease immune response and a tolerance to the antigens from the donor. Microquimerism results in the release of IL – 4, IL – 10 and transforming growth β factor; cytokines that inhibit the production of other inflammatory molecules. The loss of immunogenicity by transfused leukocytes results in anergy from T cells as well as immunosupression [11].

Different studies have reported the association between transfusions and nosocomial infection [9,12,13]. The immunomodulation generated by transfusions is a factor associated with the presence of intrahospital infections which has a great impact on mortality and costs generated from intensive care units.

Ventilator associated pneumonia (VAP) is the most common infection found in intensive care units (ICU's), with a prevalence of 47% of total infections in ICU[14]; 25% of all nosocomial infections, with an estimated 5 to 10 VAP cases per 1000 hospital admissions and 9 – 27% of intubated patients with an attributable mortality of 33% [15,16]. In addition, VAP contributes to increase morbidity due to length of stay on mechanical ventilation and hospital stay [16,17], and increasing hospital costs [18,19]. In the United States, VAP can cost approximately 40,000 dollars per case, presenting 300,000 new cases annually. Therefore, the amounts of resources used for treatment are huge; placing efforts to prevent this pathology. The main risk factor for VAP is the length of stay on mechanical ventilation increasing colonization of upper airways and stomach by pathogenic germs, further predisposing to micro aspiration which seems to be the pathophysiology for VAP [20]. Stomach colonization by pathogenic germs as a source of pneumonia is related to multiple factors such as an inadequate use of antibiotics, prophylaxis use for stress ulcers, supine position, severity of disease including hemodinamic instability and use of vasopressors; the use of continuous sedation as well as parenteral nutrition. Recognizing the risk factors might enable us to establish suitable measures for the prevention and management of VAP.

44% of the patients received one or more units of packed red blood cells during their stay in the ICU [21], these transfusions have been associated with the disruption of the immune response in the receptor [22,23] and related to the presence of severe nosocomial infections other than VAP [9,24-26]. Recently, one analysis of transfusion practice in the United States found an association between the transfusion and VAP [13]. However, no studies have been done to try and find an association between transfusions and VAP as a primary endpoint. Different trials that have found an association between nosocomial infection and transfusions were retrospective and other results came from secondary analysis or from transfusion practices. At this moment, evidence support that conservative transfusion practice in critically ill patients is as effective as the liberal strategies in terms of morbidity and mortality [27]. Unfortunately, we do not know if these practices are presently done in our ICU's. As previously stated, we consider that this study is very important and necessary to answer if there is truly a correlation between VAP and use of transfusions as well as to know the transfusion practices in our institutions. With this present study, we want to confirm the association between red blood cell transfusions and VAP as a primary endpoint. Also, identify the incidence of VAP in our ICU's establishing its characteristics, total mortality, mortality attributed to VAP and length of ICU stay as a secondary endpoint. Our theory is that if an association is found, then it is possible that by decreasing the frequency of transfusions this will decrease the incidence of VAP as well as mortality.

## Methods

### Objectives

#### Primary objective

1. Establish the association between transfusion of packed red blood cells (pRBCs) and Ventilator associated pneumonia (VAP).

#### Secondary objective

1. Describe the demographic variables in both groups.

2. Establish the incidence of VAP in both groups.

3. Establish current transfusion practices in ICU.

4. Establish the main risk factors associated with the onset of VAP in our population.

5. Estimate the incidence of anemia in patients on mechanical ventilation.

6. Describe the complications related to transfusions in our study.

### Hypothesis

#### Null hypothesis

Patients on mechanical ventilation who are transfused with packed red blood cells present an incidence of NAV equal to patients who are not transfused during the stay in ICU.

### Proposed methodology

#### Type of study

This is a prospective, multicenter, cohort study.

In the design of the study the comparison between both groups was made with a proportion of 1:1, the disparity reason of presenting VAP in patients with mechanical ventilation who were transfused is 1.89 [13] and the prevalence of VAP in the ICU is an average of 18% [16]. The sample size calculated based on the reason of disparity of the exposed group and the prevalence of the disease in the group not exposed, with an error alpha of a 0,05 and power of 0.8, the number of people that is required is of 237 patients in each arm (total of 474) to two tails. To calculate the sample we are going to use the program Epi-Info for cohort studies [28].

#### Inclusion criteria

Patients with mechanical ventilation for more than 48 hours

#### Exclusion criteria

-Younger than 18 years old.

-Pregnancy.

-Patients with contraindications for transfusion of packed red blood cells.

-Patients involved in other related investigations of transfusion practices.

-Patients with diagnosis of pneumonia within the first 48 hours of admission to ICU. (Community acquired pneumonia or nosocomial pneumonia).

### Techniques and procedures

The study will appear to the committee of ethics and investigation of the Instituto de Ciencias of Salud (CES), the financial entities to define and each institution that requires it. Then we will present to the National Network of Investigation in Mechanical Ventilation that includes ICU of Medellín, Bogota, Cali and Barranquilla.

Once the inclusion criteria are fulfilled, the patients will be followed up for 30 days, hospital discharge or death according to the first occurrence.

All the doctors will be informed about the characteristics of the study, admission criteria, measures of support and the clinical handling of the patients during their ICU stay.

### Statistical analysis

The continuous variables will be expressed as average with their standard deviation and the t Student will be used for the comparison between the averages in both groups. The categorical variables will be expressed as frequency allocations and the test of the chi-square will be used to determine the differences between the frequency allocations in both groups. A univariate analysis will be used and a multivariate logistic regression analysis to fit the independent risk factors for the development of VAP.

The respective relative risks with their corresponding confidence interval (CI) of 95% will be reported. A p value of <0.05 will be considered to determine the statistical significance. The analysis will take control of the statistical program SPSS 13. (Chicago).

### Ethical aspects

The investigation will carry out previous academic and ethical endorsements from CES and other research committees of each of the participating Clinics and Hospitals in the project. In this study the scientific norms, technical and administrative are fulfilled for the investigation in health emanated by the Ministry of Health of Colombia (resolution 8430 of 1993, by which the norms scientific, technical, and administrative settle down for the investigation in health). The investigation is based on solid scientific knowledge and it is executed by suitable medical personnel with experience in the handling of patients with this pathology.

## Discussion

The patients in the intensive care frequently are coming anemic, the etiology of this is the poor availability of iron and medullar depression by cytoquines [1], however the real impact of maintaining a good level of hematocrit in the intensive care patients is questionable, Hebert and colls in a previous trials demonstrated that a conservative management of transfusions is equal effective than a more liberal management [5].

The immunosuppressant effects of transfusion is not really studied, there are many studies that tried to made a relation between transfusion and infectious complications as a consequence of the immunosuppressant effects, Andrew Shorr and Colls as a results of a secondary analysis of the Crit study demonstrated a possible relation between ventilator associated pneumonia and transfusions[13].

Ventilator associated pneumonia constitute a primary problem in the actual management of patients in intensive care units, associated with important morbidity and attributable mortality[15,16] multiple risk factors had been associated with its presence[16].

We design a multi center prospective cohort study in multiple intensive care units in Colombia, we will follow for thirty days, patients in mechanical ventilation who do not have initial diagnosis of pneumonia.

We will describe all variables associated with pneumonia and transfusions and then after an unvaried analysis and a logistic regression analysis we will establish a really association between transfusion and pneumonia and all other factors associated with this.

Several risk factors have been associated with the occurrence of ventilator associated pneumonia, we will establish the really presence of this factors and the possible association between transfusion and ventilator associated pneumonia, also we will describe the transfusion policies in our intensive care units and the possible relation with other nosocomial infections.

## Conclusion

This is a multi center cohort study in various intensive care unit in Colombia, we will try to demonstrated as a first end point the association between pneumonia and transfusion, and all other risk factors associated with this, also we will establish the incidence of ventilator associated pneumonia and the main epidemiological factors, establish the incidence of anemia in mechanical ventilated patients and the current transfusion practice in our media.

## Competing interests

The author(s) declare that they have no competing interests.

## Authors' contributions

DY and BJ conceived the study, participated in its design, coordinated the study and drafted the manuscript, OH, RM, MG participated in the design, JPV participated in the recollection of patients. All authors read and approved the final manuscript.

## Pre-publication history

The pre-publication history for this paper can be accessed here:


